# PRaVDA: The first solid-state system for proton computed tomography

**DOI:** 10.1016/j.ejmp.2018.10.020

**Published:** 2018-11

**Authors:** Michela Esposito, Chris Waltham, Jonathan T. Taylor, Sam Manger, Ben Phoenix, Tony Price, Gavin Poludniowski, Stuart Green, Philip M. Evans, Philip P. Allport, Spyros Manolopulos, Jaime Nieto-Camero, Julyan Symons, Nigel M. Allinson

**Affiliations:** a University of Lincoln, School of Computer Science, Lincoln, UK; b University of Liverpool, Department of Physics, Liverpool, UK; c University of Warwick, Department of Physics, Warwick, UK; d University of Birmingham, School of Physics and Astronomy, Birmingham, UK; e Karolinska University, Department of Medical Physics, Stockholm, Sweden; f University of Surrey, Centre for Vision, Speech and Signal Processing, Guildford, UK; g University Hospitals Coventry and Warwickshire NHS Trust, Coventry, UK; h iThemba LABS, Somerset West, South Africa

**Keywords:** Proton therapy, Proton CT Elsevier, Solid state detectors

## Abstract

**Purpose:**

Proton CT is widely recognised as a beneficial alternative to conventional X-ray CT for treatment planning in proton beam radiotherapy. A novel proton CT imaging system, based entirely on solid-state detector technology, is presented. Compared to conventional scintillator-based calorimeters, positional sensitive detectors allow for multiple protons to be tracked per read out cycle, leading to a potential reduction in proton CT scan time. Design and characterisation of its components are discussed. An early proton CT image obtained with a fully solid-state imaging system is shown and accuracy (as defined in Section IV) in Relative Stopping Power to water (RSP) quantified.

**Method:**

A solid-state imaging system for proton CT, based on silicon strip detectors, has been developed by the PRaVDA collaboration. The system comprises a tracking system that infers individual proton trajectories through an imaging phantom, and a Range Telescope (RT) which records the corresponding residual energy (range) for each proton. A back-projection-then-filtering algorithm is used for CT reconstruction of an experimentally acquired proton CT scan.

**Results:**

An initial experimental result for proton CT imaging with a fully solid-state system is shown for an imaging phantom, namely a 75 mm diameter PMMA sphere containing tissue substitute inserts, imaged with a passively-scattered 125 MeV beam. Accuracy in RSP is measured to be ⩽1.6% for all the inserts shown.

**Conclusions:**

A fully solid-state imaging system for proton CT has been shown capable of imaging a phantom with protons and successfully improving RSP accuracy. These promising results, together with system the capability to cope with high proton fluences ($2\times 10^{8}$ protons/s), suggests that this research platform could improve current standards in treatment planning for proton beam radiotherapy.

## Highlights

• A novel proton CT imaging system, based entirely on solid-state detector technology.
• First proton CT scan with solid-state detectors.
• Potential for short scan times as well as improvement in RSP accuracy.

## 1 Introduction

Proton beam therapy (PBT), based on the use of external beams of high-energy protons, is increasingly seen as a beneficial alternative to conventional radiotherapy for some cancer treatments [1], [2]. The higher spatial selectivity of proton beams makes a stringent requirement for the accuracy that needs to be achieved in both planning and monitoring delivered dose. In fact, while the finite range of proton beams (Bragg peak) offers a highly favourable dose conformity, it also poses a substantive challenge in the prediction of delivered range to the patient and, thus, dose distribution. Several factors contribute to the uncertainty in the predicted range, including calibration to Relative Stopping Power (RSP) of X-ray CT scans used for treatment planning, anatomical changes in patients between planning and treatment, patient’s positioning errors, organ motion due to the breathing cycle during irradiation, beam reproducibility etc. [3].

An estimate of range uncertainty in proton therapy is provided by Paganetti [4], and reports a total range uncertainty of ±2.4% of the proton range plus and additional 1.2 mm. For a tumour situated at 20 cm inside a patient’s body, the uncertainty on the delivered range would be in the order of ±6 mm. This uncertainty can have a significant impact on the way dose is delivered, for example by increasing the need for larger treatment margins. Range uncertainties appear to be a major reason that prevents proton therapy reaching its maximum potential in sparing healthy tissue [3]. A number of different approaches to mitigate the effects (robust treatment planning [5]) or to reduce range uncertainties (proton radiography [6], proton computed tomography (pCT) [7], dual energy CT (DECT) [8]) are being investigated.

Several groups worldwide are working on the development of pCT imaging systems with the aim of reducing range uncertainty in treatment planning to ⩽1%, to achieve a percent dose difference (ΔD) to ‘distance to agreement’ (DTA) of ΔD/DTA = 1%/1 mm as prescribed for treatment quality assurance [9].

Two possible approaches are available for pCT, based either on proton-integrating or on proton-tracking systems. The former methodology makes use of images formed by the energy deposition of an undetermined number of incident protons, while the latter is based on the measurement of proton trajectories and energy deposition of individual protons. Although proton-integrating systems are less challenging in terms of detector performance, they are also limited by a degradation in spatial resolution, due to multiple Coulomb scattering, compared to proton-tracking systems [10], [11]. For this reason, most of the efforts in the development of pCT system are currently based on proton-tracking systems [12], [7].

Proton-tracking pCT is realised by identifying individual proton trajectories through the patient by means of tracking detectors, to which a residual energy or range can be associated. Common technological choices for pCT systems currently under development are silicon strip detectors (SSDs) [13], [14], [15] or Scintillating fibres [16], [17], [18] for the tracking system and scintillator-based calorimeters as residual energy or range detectors [13], [14], [15], [16], [17], [18], [19]. Scintillator-based calorimeters have the advantage of offering a fast readout, a direct energy measurement and an excellent energy resolution [12]. However, they are limited in terms of frame rate by their capability of tracking only a single proton per readout cycle (per segment if segmented), while segmentation poses other challenges in terms of artefacts and WEPL calibration. Position sensitive detectors appear as a promising alternative to scintillator-based calorimeters, allowing multiple protons to be tracked per readout cycle and, thus, offering a higher detection rate and a reduction in total pCT scan time. More recently, a high granularity digital tracking calorimeter based on CMOS Active Pixel Sensors (APS) has been proposed [20]. However, to date, Monte Carlo simulations and limited beam tests are available with a small prototype (19.2 × 19.2 mm^2^ active area [21]) while the integration with a tracking system, envisaged to be in the same technology, has not been addressed yet.

It is also worth mentioning that a proton-cone-beam CT system based on the use of an intensifier screen and a cooled CCD camera has been proposed [10]. However this indirect detection system is not designed to provided conventional pCT imagery, but it is based on the use of a series of proton radiographies at different energies and projection from which CT reconstruction of relative stopping power is performed.

The PRaVDA consortium was formed in 2013 to develop the first solid-state instrument for pCT, based on detector technology (SSD) developed for the ATLAS experiment at the High Luminosity Large Hadron Collider (HL-LHC, CERN), and associated novel reconstruction methods. The PRaVDA pCT system comprises two sets of trackers and a range telescope (RT). Trackers, by measuring proton entry and exit position, provide information on incoming and outgoing trajectories of individual protons, allowing reconstruction of cubic-spline paths for the protons inside the phantom/patient [22]. The RT, consisting of a stack of position sensitive detectors, allows measurement of individual proton range. Combining proton paths, as measured from trackers, with range measurements from the RT provides an estimate of energy loss by individual protons within the phantom and so an estimate of the line integral of RSP along the estimated proton track through the object.

This paper reports on the design, build and characterisation of the solid-state pCT system developed by the PRaVDA consortium. An exemplar pCT image acquired with this instrument is also shown.

## 2 Requirements and design specifications

In order to achieve high-resolution pCT images in a clinically meaningful time, it is necessary that an instrument meets the following requirements.

*High detection rate*: for a pCT scan to be acquired it is necessary to balance off the need for a large number of individual protons to be tracked (in the order of 10^9^ protons for a head CT [23]) and the strict clinical requirement to keep scan time at a reasonable length (⩽5 min). This trade-off can only be achieved with a high detection rate system combined with the capability of tracking several protons per readout cycle. This can be realised by employing position sensitive detectors, such as SSDs, read out at MHz rate.

*High detection efficiency*: in order to keep the dose to the patient as low as reasonably possible and, at the same, to limit the duration of pCT scans, high efficiency detectors are needed. SSDs are known to be more than 99% efficient for particle detection and are weakly affected by noise levels, unlike other technologies used for proton tracking in pCT such as scintillating fibres [12].

*High spatial resolution and low material budget*: to achieve high-resolution CT images that can be used in the clinical practice, sub-mm precision is required for positional and directional measurement of proton tracks. This requirement translates also into the need for low material budget (i.e. low mass detectors) to prevent multiple Coulomb scattering from deteriorating resolution performance. Such performance has been demonstrated for the PRaVDA SSDs [24].

*High WEPL resolution*: Water Equivalent Path Length (WEPL) needs to be measured with high precision to achieve sufficient image quality, while keeping dose to the patient as low as possible. WEPL resolution depends on the specific residual energy/range detector used as well as on the physics processes related to particles slowing down (i.e., *range straggling*). It has been shown [25] that, when compared to integrating energy measuring calorimeters, range counters and hybrid stage scintillators (measuring both energy and range) give an advantage in terms of WEPL resolution. Although hybrid stage calorimeters can outperform range counters, the latter offers the advantage of a simpler, faster and easier to calibrate system. The PRaVDA WEPL detector, a range telescope with 26 MHz readout, and its performance is summarised in Section 3.3.

*Radiation tolerance*: Detectors for pCT are placed directly in the beam and they need to be able to withstand high doses of radiation with unchanged performance, if they are to be used in the clinic for several years without replacement. SSDs meet the radiation requirements for extended use in clinics. SSDs used in PRaVDA have been designed for the HL-LHC and are known to provide excellent radiation tolerance to primary and secondary radiation in proton beams [26].

*Energy range and imaging area*: Ideally for proton imaging of the human body a proton beam energy as high as possible from clinical accelerators would be required (typically 230–250 MeV). Additionally, a large imaging area (e.g. 10 × 40 cm^2^) would be required to image body parts compatible with the highest energy available at clinical facilities (e.g. head, lung). For the realisation of the first PRaVDA prototype, a limited imaging area and beam energy has been used – due to limitation in terms of detector imaging area, arising from the maximum available size of 6 inch wafers in the manufacturing process. This has set design parameters for the system to be 125 MeV proton beam energy and ≈8.5 × 8.5 cm^2^ imaging area. However, it is worth noting that imaging area could be easily increased by mosaic tiling of SSDs and appropriate correction of image artefacts arising at the tiling edges, while the system could be adapted to a different beam energy by adjusting the number of detecting layers in the RT, as demonstrated with the pCT system built by the U.S. pCT collaboration [12].

## 3 The PRaVDA system

**Fig. 1:**
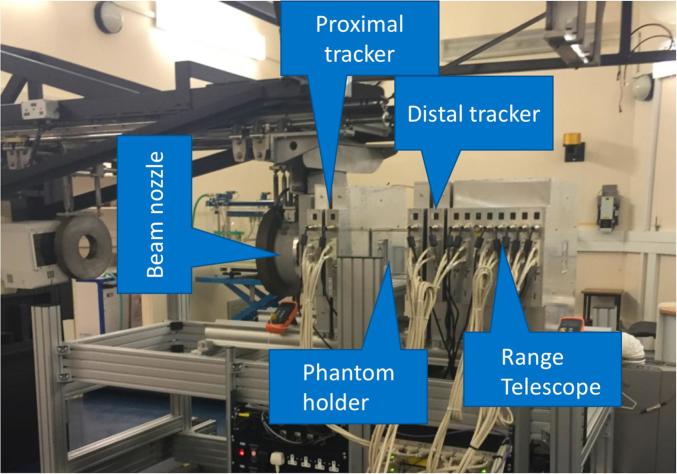
The PRaVDA pCT system showing upstream and downstream trackers, phantom holder, RT and beam nozzle at the iThemba LABS proton facility, South Africa.

The PRaVDA pCT system is shown in Fig. 1. A proximal and a distal tracker are placed before and after an imaging phantom, respectively. Trackers provide measurement for incoming and outgoing trajectories of individual protons, allowing reconstruction of proton paths inside the phantom. The RT consists of a stack of position sensitive detectors, allowing measurement of individual proton range, i.e., their residual energy.

### 3.1 Silicon strip detectors

Both trackers and RT are based on SSDs, designed by the University of Liverpool and fabricated by Micron-Semiconductor Ltd (Lancing, U.K., www.micronsemiconductor.co.uk). SSDs were made of 150 μm-thick *n-in-p* silicon with an active area of 93 × 96 mm^2^ and a strip pitch of 90.8 μm. Detectors comprise 2048 strips (channels), readout by 16 custom ASICs (Application-Specific Integrated Circuits) placed on both sides of the sensor, designed by ISDI Ltd (London, U.K.,www.isdicmos.com.) and known as RHEA (Rapid High-speed Extended ASIC). The RHEA ASICs, manufactured in a commercial 0.18 μm CMOS process, is a binary chip offering two tunable thresholds. While the low threshold is used for noise rejection, the higher threshold can be used to allow detection of double hits per channel per readout cycle, more likely at higher fluences. The ASIC is read out at a frequency of 26 MHz and up to 8 channels can be read out per readout cycle (39 ns). This translates into $2\times 10^{8}$ protons/s to be detected over the full detector area. Further details on the assembly, construction and characterisation of the PRaVDA SSDs and RHEA ASIC can be found here [27], [28].

### 3.2 Trackers

**Fig. 2:**
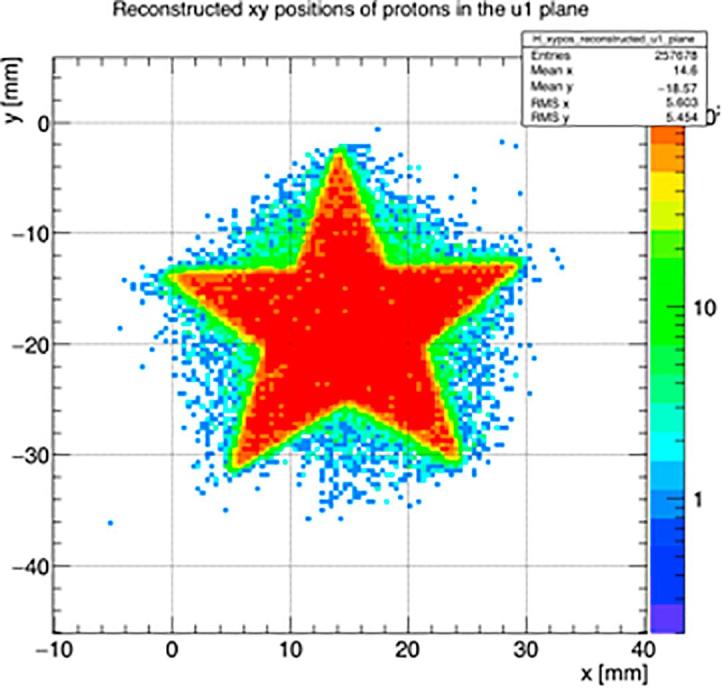
Reconstructed *x-y* coordinates for a 36 MeV proton beam imaged through a star-shaped collimator.

Each of the two PRaVDA trackers comprises 6 SSDs to form two tracking stations. In each station 3 SSDs are arranged in a so-called *x-u-v* configuration, i.e. rotated 60° to one another. Position of proton hits within each station are reconstructed by correlating positional information in each of the three planes with temporal information (timestamps) and building a virtual pixel at the crossing of three planes. Fig. 2 shows reconstructed *x-y* coordinates in a single station for a 36 MeV proton beam (MC40 Cyclotron, University of Birmingham, UK) imaged through a star-shaped collimator.

While several pCT systems use a *x-y* configuration (i.e. two detectors rotated by 90° orientations) [13], [14], [15], the chosen configuration for the PRaVDA trackers allows higher fluences to be recorded since the presence of additional positional information (i.e., extra plane) and an angle between planes <90°, reducing the fraction of ambiguous positional locations at high occupancies [29]. The vector connecting the two reconstructed positions in the proximal (distal) tracker provides the entry (exit) trajectory of individual protons crossing the phantom. Although the focus of this paper is to provide an update on the PRaVDA pCT system, it is worth mentioning that the PRaVDA trackers have been used in isolation to provide a novel pCT modality by reconstructing relative scattering power [24].

### 3.3 Range telescope

The PRaVDA RT (see Fig. 1) comprises 21 layers of SSD interleaved with 2-mm thick PMMA absorbers, providing a Water Equivalent Thickness (WET) of 2.6 mm per layer and an overall WET of 55.4 mm. The RT has been designed to stop protons in the range 30–80 MeV, as expected from a 125 MeV incident beam after passing through a 75 mm thick PMMA imaging phantom. The thickness of a single layer has been optimised to allow detection of lower energy protons, while first and last layers can be used as *veto* layers. SSDs are arranged in a 1-D configuration. A track-following algorithm, based on positional information, layer-to-layer displacement and timestamp information, has been developed to reconstruct proton tracks in the RT and to handle reconstruction of multiple tracks per readout cycle. A range value is then associated to each reconstructed proton track, corresponding to the last layer to which a track has been reconstructed.

**Fig. 3:**
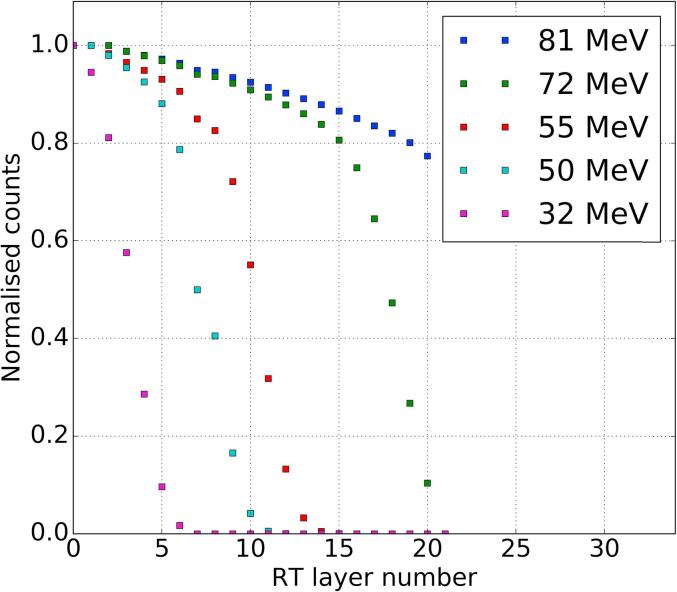
Fluence-depth curves measured with the PRaVDA range telescope for proton beams with energy in the range 32–81 MeV.

Uncertainty in measured range can be calculated, following [25], as consisting of two contributions: range straggling and uncertainty related to the thickness of each RT layer. Range straggling can be expressed as $\sigma_{s}\approx 0.011\times R_{\mathrm{tot}}$, with $R_{\mathrm{tot}}$ beam range and assuming ${\mathrm{WET}}_{\mathrm{layer}}\ll R_{\mathrm{tot}}$. The second contribution arises from the from the uncertainty of the stopping point of protons within a layer which, assuming a uniform distribution for proton range within a layer, can be written as $\sigma_{w}={\mathrm{WET}}_{\mathrm{layer}}/\sqrt{12}$. The total range uncertainty will then be: $\sigma_{r}=\sqrt{\sigma_{s}^{2}+\sigma_{w}^{2}}$. For our experiment $\sigma_{r}=1.5$ mm or 1.3% of the beam range. Capabilities of range measurement are shown in Fig. 3, where fluence-depth curves are shown for a number of proton energies. A 125-MeV proton beam (iThemba LABS, South Africa) was degraded by insertion of PMMA absorbers between proximal and distal trackers to produce beam energies in the range 32–81 MeV. Normalised number of protons (counts) is shown as a function of layer number in the RT. Fluence-depth curves of Fig. 3 show, as expected, a gradual decline – due to inelastic collision of protons with atomic nuclei – followed by a sharp drop which corresponds to the proton range. For the energies shown in Fig. 3, a decrease in range with proton energy can be seen.

### 3.4 Data acquisition system

The PRaVDA custom data acquisition system (DAQ) was designed and manufactured by aSpect Systems GmbH (Dresden, Germany.http://www.aspect-sys.com). It is based on a highly modular design, which provides flexibility to seamlessly adjust the instrument to different experimental conditions (proton energy, phantom, thickness etc.) by simply adding or removing readout modules. Each module represents a group of 3 SSDs and their associated FPGAs, local memory and internal multiplexer, whose data output is handled by an external multiplexer. Data streams from each readout module is managed by a third level of multiplexers. The total data rate for the PRaVDA system is 28 Gb/s for the trackers and 42 Gb/s for the RT, with a combined data rate of 66 Gb/s.

## 4 Results

**Fig. 4:**
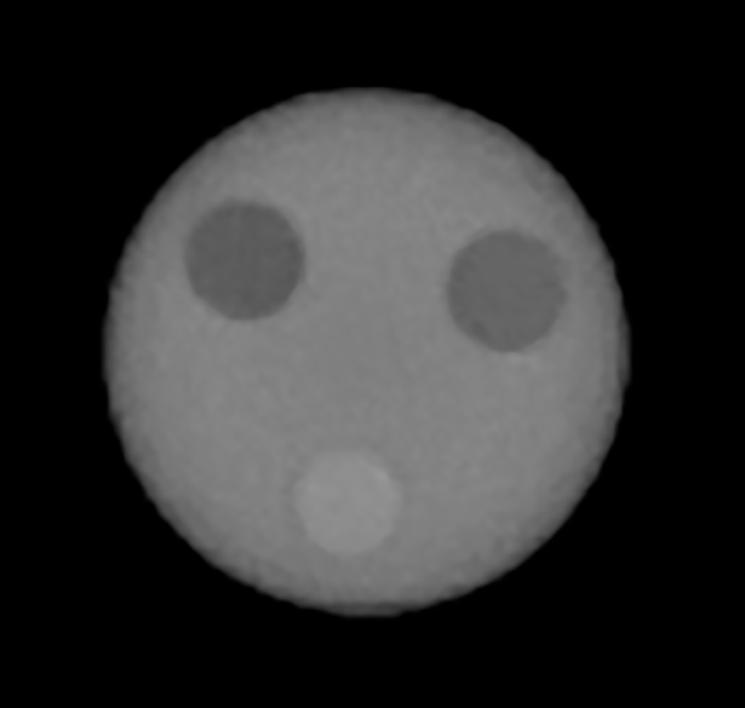
A pCT slice for a spherical phantom containing 3 substitute inserts (top left: water equivalent, top right: adipose equivalent, bottom: average bone equivalent).

An exemplar proton CT transverse slice obtained using the PRaVDA system is shown in Fig. 4. A PMMA spherical phantom of 75 mm diameter containing tissue substitute inserts (cylindrical with a 15 mm diameter) was imaged using a 125 MeV proton beam (85 mm diameter) at iThemba LABS, SA. A range compensator, i.e. a 75 mm cube from which a 75 mm diameter sphere had been removed, was placed before the proximal tracker to reduce the range spread. One hundred and eighty projections, with each projection requiring 1 s to acquire, were acquired over 360° and a total of $2.8\times 10^{8}$ proton histories were tracked and their range, calibrated in WEPL, measured. CT reconstruction was then performed using a back-projection-then-filtering algorithm (BPF) [22]. The image shows a reconstructed slice containing the following tissue substitute inserts: adipose equivalent, average bone equivalent and water equivalent^1^.

A direct measurement of the RSP of the tissue substitute inserts was performed using the range-shift method for different samples of the same material, and compared with the RSP measured in a pCT slice (calculated as average RSP across a ROI of approximately 50 pixels). RSP values for the 3 tissue substitute inserts of Fig. 4 are reported in Table 1. The difference in RSP from direct measurement and derived from the proton CT (RSP accuracy) was −0.7, 1.2 and 1.6% for the adipose equivalent, average bone and water equivalent inserts, respectively.

**Table 1:** Expected RSP measured using the range-shift method, pCT RSP, obtained from the pCT slice of Fig. 4 and their relative difference (RSP accuracy).

Material	Expected RSP	pCT RSP	RSP accuracy
			%
Adipose	0.95	0.94	0.7
Average bone	1.21	1.22	1.2
Water	1.00	0.98	1.6

## 5 Conclusions

The first fully solid-state imaging system for pCT has been presented. Design and performance of trackers and RT, both based on SSD detector technology, have been discussed and their capabilities in proton tracking and range measurement demonstrated. The position sensitive detectors used in this instrument, together with its tens MHz readout, allow for a fast pCT scan with $2\times 10^{8}$ protons/s detectable over the full imaging area. A pCT image obtained with this system has been shown and accuracy in RSP for several tissue substitute inserts quantified as ⩽1.6%. Potential for short scan times as well as improvement in RSP accuracy, when compared to conventional CT, highlight the potential for the PRaVDA imaging system to improve current standards in treatment planning for PBT.

The results presented here are interim and can be refined further. PRaVDA was not intended as prototype pCT system that could be directly transformed in a clinical instrument. It was a research test-rig to fully understand the potential of solid-state sensors to provide very high count rates and precision measurements as a precursor to the next stage of pre-commercial prototyping.

## Footnotes

1 These materials were samples of the so-called Barts materials and were supplied by Leeds Test Objects (Boroughbridge, UK). Composition codes were: AP7 (adipose equivalent), RB2 (average bone equivalent) and WT1 (water equivalent).
